# Supplementary material for: Predicted glycosyltransferases promote development and prevent spurious cell clumping in the choanoflagellate S. rosetta
Source: eLife. 2018 Dec 17;7:e41482. doi: 10.7554/eLife.41482 (PMC6322860; doi:10.7554/eLife.41482)
Supplement: Supplementary file 1. — (1) Table S1. Phenotypic classes of mutants isolated in this study and in the Levin et al. (2014) screen. (2) Table S2. Segregating variants in Rosetteless mapping cross. (3) Table S3. Segregating variants in Jumble mapping cross. (4) Table S4. Segregating variants in Couscous mapping cross. (5) Table S5. Fluorescent lectins tested. [file elife-41482-supp1.docx]

| **Table S1. Phenotypic classes of mutants isolated in this study and in the Levin *et al.* 2014 screen.** | | | | | | |
| --- | --- | --- | --- | --- | --- | --- |
| **Mutant Class** | **Strain** | **Mutagen** | **Single cell interactions** | **Rosette morphology** | **% cells in rosettes** | |
|  |  |  |  |  | **Live *Algori-phagus*** | **OMVs** |
|  | wt | - | Non-clumping | wt | 87 | 95.2 |
| Class A | Rosetteless^†^ | EMS | Non-clumping | n.d. | 0 | 0 |
|  | M7G9 | X-rays | Non-clumping | n.d. | 0 | 0 |
|  | M1A1.F3 | Spontaneous | Non-clumping | n.d. | 0 | 0 |
| Class B | Insensate^†^ | X-rays | Non-clumping | Irregular | 3.1 | 5.6 |
|  | Slacker^†^ | X-rays | Non-clumping | Irregular | 42.4 | nt |
|  | Uptight^†^ | X-rays | Non-clumping | Irregular | 53.1 | nt |
|  | M14A9.D5 | X-rays | Non-clumping | Irregular | 56.9* | 76.3* |
|  | M17C12 | X-rays | Non-clumping | Irregular | nt | 24* |
| Class C | Jumbled^†^ | EMS | Clumping | n.d. | 0 | 0 |
|  | Couscous^†^ | X-rays | Clumping | n.d. | 0 | 0 |
|  | Seafoam^†^ | X-rays | Clumping | n.d. | 0 | 0 |
|  | Soapsuds^†^ | X-rays | Clumping | n.d. | 0 | 0 |
|  | M5G11.E8 | X-rays | Mild clumping | n.d. | 0 | 0 |
|  | M13H12.G2 | X-rays | Mild clumping | wt | 5.2 | 4 |
|  | M1C5.D2 | X-rays | Mild clumping | wt | 0 | 37* |
| Class D^‡^ | Solo | X-rays | Non-clumping | No rosettes | 0 | nt |
| ^†^Originally reported in Levin *et al.,* 2014; n.d.= not detected; nt= not tested; * ≤ 2 biological replicates; ^‡^Class D mutant fails to form chains in the absence of RIFs and is therefore distinct from Class A mutants. | | | | | | |

| **Table S2. Segregating variants in Rosetteless mapping cross** | | | | | |  |
| --- | --- | --- | --- | --- | --- | --- |
| Supercontig | Location | Position relative to genes | Type | Coverage^†^ | | |
| 4 | 516,051 | intron | INDEL* | 8 | | |
| ­­6 | 1,139,589 | 5’ UTR | INDEL* | 40 | | |
| **8** | **427,804** | **splice donor** | **SNV**** | **253** | | |
| 11 | 524,974 | intron | INDEL* | 12 | | |
| 11 | 1,660,350 | intron | INDEL* | 6 | | |
| **Average genome-wide coverage^†^: 187** | | | | |  |  |
| ^†^Number of high quality reads determined by SAMtools (Li et al., 2009) at nucleotide position; *Insertion or deletion; **Single nucleotide variant; Highlighted sequence variant indicates known causative lesion (Levin et al., 2014). | | | | |  |  |

| **Table S3. Segregating variants in Jumble mapping cross** | | | | |
| --- | --- | --- | --- | --- |
| Supercontig | Location | Position relative to genes | Type | Coverage^†^ |
| **1** | **1,919,681** | **coding sequence** | **SNV**** | **165** |
| 20 | 530,561 | intron | INDEL* | 3 |
| 22 | 65,983 | intron | INDEL* | 37 |
| 32 | 134,832 | intron | INDEL* | 5 |
| 49 | 3,863 | intron | SNV** | 2 |
| **Average genome-wide coverage^†^: 187** | | | | |
| ^†^Number of high quality reads determined by SAMtools (Li et al., 2009) at nucleotide position; *Insertion or deletion; **Single nucleotide variant; Highlighted sequence variant indicates predicted causative lesion. | | | | |

| **Table S4. Segregating variants in Couscous mapping cross.** | | | | |
| --- | --- | --- | --- | --- |
| Supercontig | Location | Position relative to genes | Type | Coverage^†^ |
| 3 | 1,812,030 | splice acceptor | INDEL* | 2 |
| 4 | 475,982 | intron | INDEL* | 10 |
| 4 | 518,253 | intron | INDEL* | 12 |
| 5 | 533 | intron | INDEL* | 3 |
| 9 | 141,246 | intron | INDEL* | 3 |
| 13 | 698,752 | intron | INDEL* | 6 |
| 22 | 110,265 | intron | INDEL* | 5 |
| **22** | **462,534** | **coding sequence** | **INDEL*** | **128** |
| **Average genome-wide coverage^†^: 72** | | | | |
| ^†^Number of high quality reads determined by SAMtools (Li et al., 2009) at nucleotide position; *Insertion or deletion; Highlighted sequence variant indicates predicted causative lesion. | | | | |

| **Table S5. Fluorescent lectins tested** | | | | | | | |  |
| --- | --- | --- | --- | --- | --- | --- | --- | --- |
| Lectin | | Preferred Sugar Specificity* | wild type Localization | | Jumble  Localization | | Couscous  Localization | |
| Con A (Concanavalin A) | | αMan, αGlc | Bacteria | | Bacteria | | Bacteria | |
| SBA (*Glycine max*(soybean) agglutinin) | | α>βGalNAc | Faint cell body | | Faint cell body | | Faint cell body | |
| DBA (*Dolichos biflorus* agglutinin) | | αGalNAc | Faint cell body | | Faint cell body | | Faint cell body | |
| DSL (*Datura Stramonium*lectin) | | (GlcNAc)_2-4_ | n.d. | | n.d. | | n.d. | |
| ECL (*Erythrina cristagalli*lectin) | | Galβ4GlcNAc | n.d. | | n.d. | | n.d. | |
| GSL I (*Griffonia (Bandeiraea) simplicifolia* lectin I) | | αGal, αGalNAc | Patchy cytoplasmic | | Patchy cytoplasmic | | Patchy cytoplasmic | |
| GSL II (*Griffonia (Bandeiraea) simplicifolia*lectin II) | | α or βGlcNAc | n.d. | | n.d. | | n.d. | |
| **Jacalin** | | **Galβ3GalNAc** | **Basal pole and collar base** | | **Collar base** | | **Collar base** | |
| LCA (*Lens culinaris* agglutinin) | | αMan, αGlc | n.d. | | n.d. | | n.d. | |
| LEL (*Lycopersicon esculentum*(tomato) lectin) | | (GlcNAc)_2-4_ | Collar base | | Collar base | | Collar base | |
| MAL I (*Maackia Amurensis* lectin I) | | Galβ4GalNAc | Cell membrane | | Cell membrane | | Cell membrane | |
| PHA-E (*Phaseolus vulgaris*Erythroagglutinin) | | Galβ4GlcNAcβ2Manα6  (GlcNAcβ4)  (GlcNAcβ4Manα3)  Manβ4 | Faint cell body | | Faint cell body | | Faint cell body | |
| PHA-L (*Phaseolus vulgaris*Leucoagglutinin) | | Galβ4GlcNAcβ6(GlcNAc  β2Manα3)Manα3 | n.d. | | n.d. | | n.d. | |
| PNA (*Arachis hypogaea*(peanut) agglutinin) | | Galβ3GalNAc | n.d. | | n.d. | | n.d. | |
| PSA (*Pisum sativum* Agglutinin) | | αMan, αGlc | Faint cell body | | Faint cell body | | Faint cell body | |
| RCA_120_ (*Ricinus communis* agglutinin) | | Gal | Cell body | | Cell body | | Cell body | |
| SNA (*Sambucus Nigra* Lectin) | | Neu5Acα6Gal/GalNAc | n.d. | | n.d. | | n.d. | |
| STL (*Solanum tuberosum*(potato) lectin) | | (GlcNAc)_2-4_ | Collar base | | Collar base | | Collar base | |
| Succinylated WGA (Wheat germ agglutinin, succinylated) | | GlcNAc | Cell membrane | | Cell membrane | | Cell membrane | |
| UEA I (*Ulex europaeus*agglutinin I) | | αFuc | n.d. | | n.d. | | n.d. | |
| VVL (*Vicia villosa*agglutinin) | | GalNAc | Faint cell body | | Faint cell body | | Faint cell body | |
| WGA (*Triticum vulgaris (*wheat germ) agglutinin) | | GlcNAc | Cell membrane | | Cell membrane | | Cell membrane | |
| *From Vector Laboratories Product Information | | | | | | | |  |
| Symbols and abbreviations: | | | | | | | | |
| n.d.  >  Fuc  Gal  GalNAc | not detected  preference for first over second sugar  L-Fucose  D-Galactose  *N­*-Acetylgalactoamine | | | Glc  GlcNAc  Man  Neu5Ac | | D-Glucose  *N*-Acetyleglucosamine  Mannose  N-Acetylneuraminic acid (sialic acid) | | |

­­­­

**SUPPLEMENTARY REFERENCES**

Levin TC, Greaney AJ, Wetzel L, King N. 2014. The *rosetteless* gene controls development in the choanoflagellate *S. rosetta*. *eLife* **3**:e04070. doi:10.7554/eLife.04070

Li H, Handsaker B, Wysoker A, Fennell T, Ruan J, Homer N, Marth G, Abecasis G, Durbin R. 2009. The Sequence Alignment/Map format and SAMtools. *Bioinformatics* **25**:2078–2079. doi:10.1093/bioinformatics/btp352
